# Supplementary material for: Control of Organ Abscission and Other Cell Separation Processes by Evolutionary Conserved Peptide Signaling
Source: Plants (Basel). 2019 Jul 15;8(7):225. doi: 10.3390/plants8070225 (PMC6681299; doi:10.3390/plants8070225)
Supplement: Supplementary file 1 [file plants-08-00225-s001.pdf]

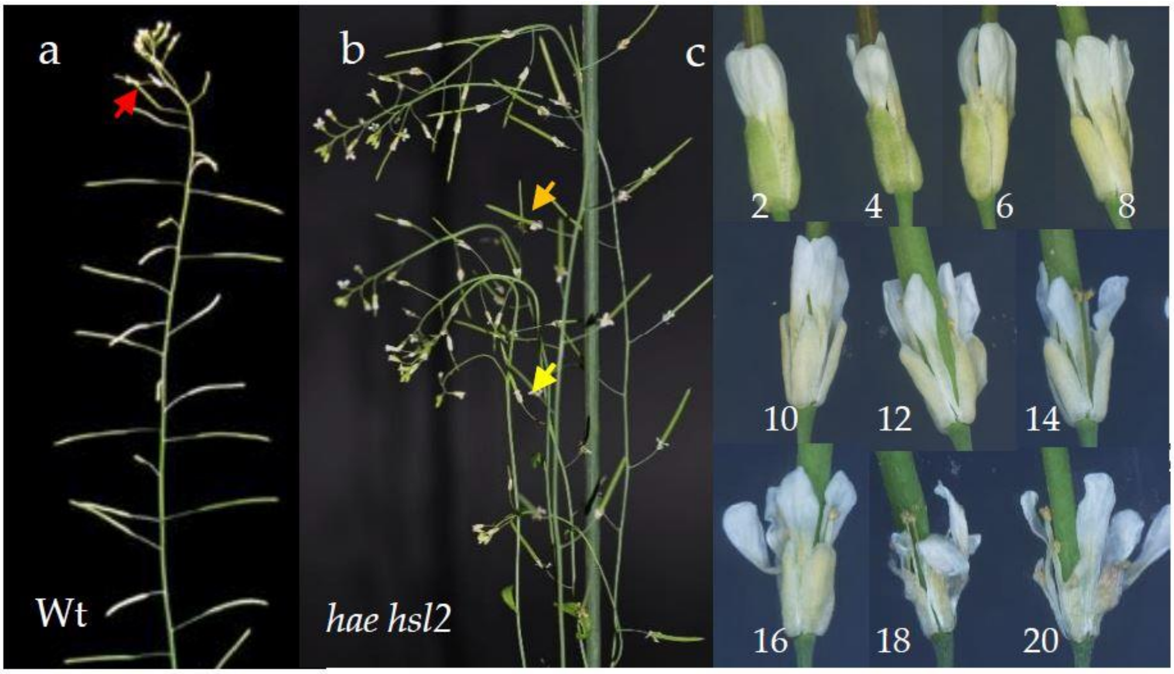


**Figure S1.** The double mutant *hae hsl2* is totally deficient in abscission. **(a)** Inflorescence of wild type Arabidopsis plant. Red arrow points to first siliques with absciced floral organs. **(b)** *hae hsl2* double mutant. Orange and yellow arrows indicate silique positions 16 and 20, respectively, counting from the top of the inflorescense. **(c)** Attached floral organs of the *hae hsl2* double mutant are turgid at least until position 16.

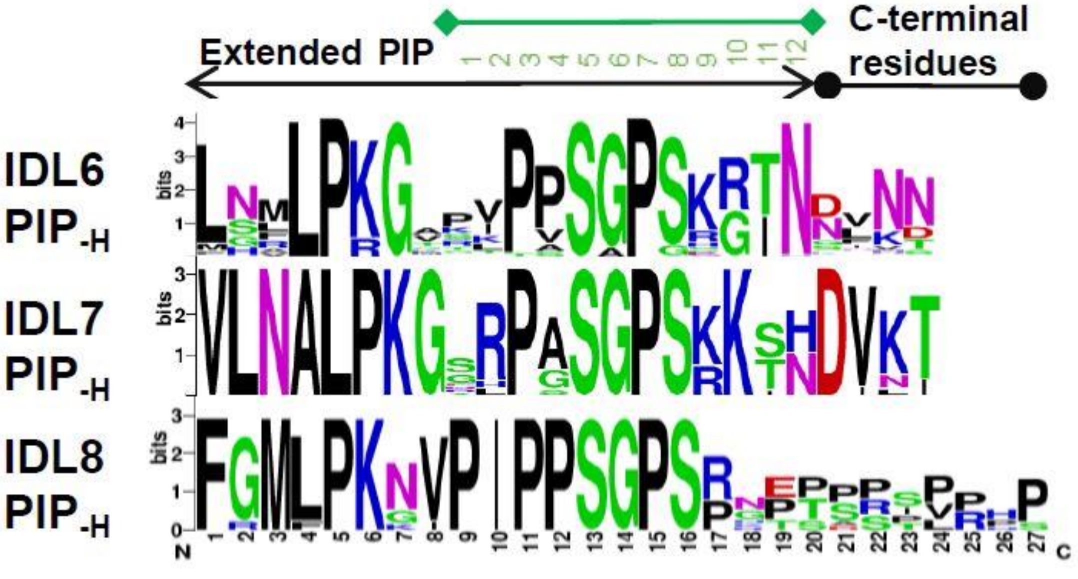


**Figure S2.** Conserved residues in propeptides of angiosperm IDL 6 8 orthologues numbered from the start of the extended PIP motif to the C terminal end. Sequences were aligned using Muscle, and consensus sequences were generated with logo@compbio berkeley edu The PIP residues are numbered 1 12 Compared to IDA and IDLA 1 5 IDL 6 8 lack the His ( in position 11 The Arg His Asn ( of IDA has been shown to interact with the co receptor SERK 1 22.

| LRR | | *Arabiopsis thaliana* **HAE**<br>**LxxLxxLxLxxNxLSGx-IPxx-LGx** | | *Arabidopsis thaliana* **HSL2**<br>**LxxLxxLxLxxNxLSGxIPxx-LGx** | | *Elais guineensis* **EgHSL2**<br>**LxxLxxLxLxxNxLS-GxIPxxLGx** |
|---|---|---|---|---|---|---|
| 1 | 88 | LPSLHSLSLYNNSINGS-LSADDFDT | 97 | IRTLINITLSQNNLNGTIDSAPLSL | 121 | IPTLRHLSLAWNDLGGGLPAADVAL |
| 2 | 113 | CHNLISLDLSENLLVGS-IPKSLPFN | 122 | CSKLQNLILNQNNFSGKLPEF-SPE | 118 | CSGLEVLNLSNNFFV-GRLPEFSGE |
| 3 | 138 | LPNLKFLEISGNNLSDT-IPSS-FGE | 146 | FRKLRVLELESNLFTGEIPQS-YGR | 142 | FPRLRLLDLSGNNFS-GDIPPSFGR |
| 4 | 162 | FRKLESLNLAGNFLSGT-IPAS-LGN | 170 | LTALQVLNLNGNPLSGIVPAF-LGY | 166 | FPSLRVLSLYSNLIS-GRIPSFLAN |
| 5 | 186 | VTTLKELKLAYNLFSPSQIPSQ-LGN | 190 | LTELTHLDLAYISDPSPIPST-LGN | 190 | LTELIEFNLGFNPFRSGSLPPEIGN |
| 6 | 211 | LTELQVLWLAGCNLVGP-IPPS-LSR | 219 | LSNLTDLRLTHSNLVGEIPDS-IMN | 215 | LTKLEVLWLPFVNLV-GEIPDSVGN |
| 7 | 235 | LTSLVNLDLTFNQLTGS-IPSW-ITQ | 243 | LMLLENLDLAMNGLTGEIPES-IGT | 239 | LANLKILDLSNNGLT-GRIPASIGR |
| 8 | 259 | LKTVEQIELFNNSFSGE-LPES-MGN | 267 | LESVYQIELYDNRLSGKLPES-IGN | 263 | LRSVEKMELWRNQLS-GELPQSLGN |
| 9 | 283 | MTTLKRFDASMNKLTGK-IPDN-LN- | 291 | LTELRNFDVSQNNLTGELPEK-IA- | 287 | LTSLFAFDASENKLT-GKLPE-GLA |
| 10 | 306 | LLNLESLNLFENMLEGP-LPES-ITR | 314 | ALQLISFNLNDNFFTGGLPDV-VAL | 310 | GLNLTSLALNDNRMD-GEISTVLAQ |
| 11 | 330 | SKTLSELKLFNNRLTGV-LPSQ-LGA | 338 | NPNLVEFKIFNNSFTGTLPRN-LGK | 334 | NPHLVELKLFNNNFS-GELPSGLGR |
| 12 | 354 | NSPLQYVDLSYNRFSGE-IPAN-ICG | 362 | FSEISEFDVSTNRFSGELPPY-LCY | 358 | YSYLTNVDVSGNQFA-GRLPPDLCS |
| 13 | 378 | EGKLEYLILIDNSFSGE-ISNN-LGK | 386 | RRKLQKIITFSNQLSGEIPES-YGD | 382 | RGMLESLVAFGNRFS-GELPQSYAD |
| 14 | 402 | CKSLTRVRLSNNKLSGQ-IPHGFWG- | 410 | CHSLNYIRMADNKLSGEVPAR-FWE | 409 | CRTLDYVRIQNNELS-GKVPDLFWS |
| 15 | 426 | LPRLSLLELSDNSFTGS-IPKTIIG- | 434 | LPLTRLELANNNQLQGSIPPS-ISK | 430 | LPKLYHLELRGNKLE-GSLPPNISR |
| 16 | 450 | AKNLSNLRISKNRFSGS-IPNE-IGS | 458 | ARHLSQLEISANNFSGVIPVK-LCD | 454 | AKNLTQIIISDNKFS-GQIPPEICD |
| 17 | 474 | LNGIIEISGAENDFSGE-IPES-LVK | 482 | LRDLRVIDLSRNSFLGSIPSC-INK | 478 | LMELRTFDAGNNQFS-SGLPLCIAD |
| 18 | 498 | LKQLSRLDLSKNQLSGE-IPRE-LRG | 506 | LKNLERVEMQENMLDGEIPSS-VSS | 502 | LTKLQVLDLQQNNFS-GEIPA-GG- |
| 19 | 522 | WKNLNELNLANNHLSGE-IPKE-VGI | 530 | CTELTELNLSNNRLRGGIPPE-LGD | 524 | WTELAEINLSMNRFS-GEIPRSLGD |
| 20 | 546 | LPVLNYLDLSSNQFSGE-IPLE-LQN | 554 | LPVLNYLDLSNNQLTGEIPAE-LLR | 548 | LPVLTYLDLSGNQLS-GEIPPELTN- |
| 21 | 570 | L-KLNVLNLSYNHLSGK-IPPL-YA- | 578 | L-KLNQFNVSDNKLYGKIPS--FQ- | 572 | L-KLNHLNLSGNDLS-GKIPD--GF- |
| 22 | 592 | NKIYAHDFIGNPGLCVD-LDG--LCR | 600 | QDIFRPSFLGNPNLCAPNLDPIRPC | 594 | DTSFFLPSLLGNPDLCSSSGLKTFRRC |

**Figure S3.** Structure-based sequence alignment of the 21 leucine-rich repeats (LRRs) comparing Arabidopsis AtHAE and AtHSL2 with EgHSL2 from oil palm. Residue numbers are found in the columns to the left of the respective alignments. The plant LRR consensus sequence making up the scaffold of the ectodomain is shown on top of the alignments and is shaded in grey throughout the LRRs. Residues shown from the AtHAE-AtIDA crystal structure to mediate hydrophobic interactions with AtIDA, and corresponding conserved residues in AtHSL2 and EgHSL2, are highlighted in blue, residues contributing to hydrogen bond interactions and/or salt bridges are shown in red. N-glycosylation sites are indicated in green, cysteine residues involved in disulphide bridge formation in orange, and residues involved in binding of AtSERK1 in yellow.
